# Supervision and feedback for junior medical staff in Australian emergency departments: findings from the emergency medicine capacity assessment study

**DOI:** 10.1186/1472-6920-10-74

**Published:** 2010-11-02

**Authors:** George A Jelinek, Tracey J Weiland, Claire Mackinlay

**Affiliations:** 1 Emergency Department, St Vincent's Hospital, Fitzroy, Victoria, Australia; 2 Department of Medicine, University of Melbourne, Fitzroy, Victoria, Australia

## Abstract

**Background:**

Clinical supervision and feedback are important for the development of competency in junior doctors. This study aimed to determine the adequacy of supervision of junior medical staff in Australian emergency departments (EDs) and perceived feedback provided.

**Methods:**

Semi-structured telephone surveys sought quantitative and qualitative data from ED Directors, Directors of Emergency Medicine Training, registrars and interns in 37 representative Australian hospitals; quantitative data were analysed with SPSS 15.0 and qualitative data subjected to content analysis identifying themes.

**Results:**

Thirty six of 37 hospitals took part. Of 233 potential interviewees, 95 (40.1%) granted interviews including 100% (36/36) of ED Directors, and 96.2% (25/26) of eligible DEMTs, 24% (19/81) of advanced trainee/registrars, and 17% (15/90) of interns. Most participants (61%) felt the ED was adequately supervised in general and (64.2%) that medical staff were adequately supervised. Consultants and registrars were felt to provide most intern supervision, but this varied depending on shift times, with registrars more likely to provide supervision on night shift and at weekends. Senior ED medical staff (64%) and junior staff (79%) agreed that interns received adequate clinical supervision. Qualitative analysis revealed that good processes were in place to ensure adequate supervision, but that service demands, particularly related to access block and overcrowding, had detrimental effects on both supervision and feedback.

**Conclusions:**

Consultants appear to provide the majority of supervision of junior medical staff in Australian EDs. Supervision and feedback are generally felt to be adequate, but are threatened by service demands, particularly related to access block and ED overcrowding.

## Background

Clinical supervision, education and feedback are critical to the development of competency in the junior doctor. Supervision has the combined objectives of preventing mistakes, poor practice and adverse events, while facilitating opportunistic and "practice-based" learning together with a degree of junior doctor autonomy [1]. Increased supervision is linked with improved junior doctor performance and better patient outcomes [2]. With increasing demands on our health system, recognising the importance of providing and sustaining quality supervision, education and feedback in post-graduate medical training will be fundamental to developing and maintaining a quality healthcare system.

Emergency departments offer a unique educational environment for the developing doctor. Patients present to EDs in large numbers, often undifferentiated, and with diverse conditions. There are great opportunities to learn procedures, and gain an overview of the health system. However, a potential threat to the provision of supervision, education and feedback to junior ED staff is a lack of availability of senior staff to supervise. In response to the shortfall in medical personnel in Australia the Government has funded additional medical school places which will result in up to a 90% increase in graduate numbers. This influx will peak in 2012 and is likely to result in increased supervisory demands in the context of an already overstretched service. Thus the antidote to the workforce shortage may provoke a demand for supervision that is difficult to meet.

It has been argued that clinical supervision is inadequate in Australia as it is in the United Kingdom [3], although there are little data on which to base this, certainly in the ED. The education and supervision experienced by prevocational doctors and interns in the ED has been investigated in several surveys. Registrars appear to do the bulk of supervision and training [4,5]. In one study, 90% of prevocational doctors reported adequate informal contact with registrars, but only 56% reported adequate informal contact with consultants [4]. Other studies have yielded similar results [5,6].

The level of assessment and feedback provided to interns during their ED rotation is another area requiring attention. Mid-term and end of term formal feedback by supervisors is a general requisite of accrediting postgraduate councils [7]. However, it has been suggested that in most hospitals there is varying compliance with this [7].

In 2008, St. Vincent's Hospital Melbourne was contracted by the Commonwealth of Australia's Department of Health and Ageing to conduct a capacity analysis of Emergency Departments (EDs) in light of the projected large increase in interns graduating from Australian medical schools, peaking in 2012. Using telephone survey methodology, this Emergency Medicine Capacity Assessment Study (EMCAS) sought to characterise issues related to the capacity and strategies of EDs and staff to support increasing numbers of medical graduates.

The current marked increase in junior medical staff in EDs as part of the planned workforce expansion in Australia may place considerable strain on ED supervision and feedback processes. To date, however, data regarding the adequacy of supervision and feedback as perceived by ED medical staff have been lacking. It is important to understand current supervision and feedback arrangements for junior medical staff in Australian EDs to inform planning for managing the anticipated workforce expansion. Using a mixed-methods approach we aimed to explore the perceptions of three key staff groups, ED Directors, registrars and interns regarding the adequacy of current supervision of junior staff in this setting and the form and perceived adequacy of the feedback provided to them.

## Methods

The methodology has previously been described in detail [8]; briefly, the study was conducted as follows.

### Project oversight

The project was devised and run by St. Vincent's Hospital Melbourne, with a Steering Committee reviewing telephone survey questions, pilot data collection, national data collection and data analysis phases.

### Design

Data for this study were collected using an exploratory design involving surveys generating qualitative and quantitative data with open and forced categorical responses.

### Surveys

Semi-structured telephone survey was used to reach the large dispersed target group. Survey questions were developed by an emergency physician with input from a researcher/psychologist and a project officer experienced in survey methodology and workforce planning. Face validity of draft survey items was ensured through feedback between project staff and the steering committee. Refinement of questions and methodology was undertaken using a pilot sample of doctors from St. Vincent's Hospital. Data from the pilot were excluded from final analyses.

The final overall EMCAS survey schedule comprised 160 items for ED Directors/Directors of Emergency Medicine Training (DEMT), and up to 97 items for registrar and intern participants. Of these, 40/160 items for ED Directors/DEMT and 24/97 items for registrar and intern participants concerned supervision and feedback. The data items sought graded responses using Likert scales or ordinal multi-category scales to enable quantitative statistical analysis. Questions were asked specifically related to: adequacy of supervision of the ED and of ED staff, and in particular of interns; and of feedback provided to interns and other junior medical staff, including open-ended questions. A definition of "adequacy" was not provided to participants.

### Nationwide Study

Doctors were eligible for participation if they were Emergency Department Directors, DEMT, advanced trainees in EM, or prevocational doctors (interns) who had completed at least half their EM rotation. For the larger Australian States, participants were drawn from a stratified sample of two city, two metropolitan and two provincial hospitals of each State/Territory. Hospitals were selected based on the advice of the relevant postgraduate medical councils who were asked to nominate hospitals with EDs and prevocational doctors "representative" of the state for each category (city, metropolitan, provincial). As there were insufficient hospitals in the ACT, TAS, NT to meet our sampling criteria all hospitals with applicable EDs from these regions were included. The total number of target hospitals was 37.

### Recruitment and Data collection

Coordinators at each site distributed an email invitation to eligible participants. Those agreeing were emailed a participant information statement and the full survey schedule to consider their responses prior to survey. All participants provided informed consent. Telephone surveys were administered by one researcher (apart from one survey by a second trained researcher during a period of absence) and responses were recorded to permit verbatim transcription; voice recordings were stored electronically. Recordings were later replayed and data entered into a predefined electronic survey form. Separate though similar surveys were administered to the three groups: (1) ED Directors and DEMT; (2) advanced trainees in EM, and (3) interns.

### Sample size

Since the purpose of the study was to describe rather than compare responses for different groups using inferential statistics no sample size calculations were performed.

### Data Analysis

Data entered into the web-based survey program were exported to Microsoft Excel; de-identified data were analysed using SPSS 15.0. The response distribution for quantitative items was summarised using frequencies, percentages ± 95% CI and median as appropriate. The total number of graded responses analysed varied for each item as some respondents were unable to answer all questions. These data are presented as percentage calculations adjusted for missing data. Open-ended questions yielding qualitative data were subjected to content analyses by two researchers and inter-rater reliability was verified using Cohens' unweighted kappa. Themes were identified using the method of Ritchie and Spencer [9] and the final categorisation of themes used those provided by a single coder who is an emergency physician.

### Ethics

This study was approved by the Ethics Committees responsible for the 36 participating sites around Australia, as follows (some committees approved the study for more than one hospital site): ACT Health and Community Care Human Research Ethics Committee, Ballarat Health Services & St John of God Health Care Ethics Committee, Cairns Base Hospital Ethics Committee, Calvary Health Care ACT Human Research Ethics Committee, Central Australian Human Research Ethics Committee, Central Northern Adelaide Health Services Ethics of Human Research Committee (TQEH & LMH), Eastern Health Research and Ethics Committee, Flinders Clinical Research Ethics Committee, Gold Coast Health Service District Human Research Ethics Committee, Goulburn Valley Health Ethics and Research Committee, Greater Southern Area Health Service HREC, Human Research Ethics Committee - A St Vincent's Health Melbourne, Human Research Ethics Committee of Northern Territory Department of Health & Community Services, Hunter New England Human Research Ethics Committee, King Edward Memorial Hospital for Women Ethics Committee, Mackay Health Service District Human Research Ethics Committee, Melbourne Health Human Research Ethics Committee, Monash University Standing Committee on Ethics in Research Involving Humans, Northern Sydney Health Human Research Ethics Committee (Hawkesbury), Princess Alexandra Hospital Human Research Ethics Committee, Redcliffe-Caboolture Health Service District Ethics Committee, Royal Adelaide Hospital Research Ethics Committee, Royal Brisbane and Women's Hospital HREC, Royal Perth Hospital Ethics Committee, SA Department of Health Human Research Ethics Committee, South Eastern Sydney and Illawarra Area Health Service HREC - Northern Sector, South Western Sydney Area Health Service - Human Research Ethics Committee, Tasmania Health & Medical Human Research Ethics Committee, WA Country Health Service Board Research Ethics Committee, Women's & Children's Hospital Research Ethics Committee.

## Results

### Participation

As described previously^8 ^participants were drawn for 36 of 37 target hospitals. A total of 95 participants completed the survey including 36 ED Directors (or Director/DEMTs) representing 36 hospitals, 25 DEMT representing 25 hospitals; 19 registrars representing 12 hospitals and 15 interns representing 12 hospitals.

For sites with a low or no response rate, site co-ordinators sent up to two repeat invitations to eligible staff. The duration of surveys ranged from 20-90 minutes with an average of 40 minutes. Survey duration was primarily affected by the length of responses to open-ended questions. Several participants were offered a break during the survey however none was taken.

### Content analysis

The kappa value for the inter-rater reliability of content analysis by the two researchers analysing open-ended questions was 0.80 (n = 89 responses).

### Supervision

**Figure 1:**
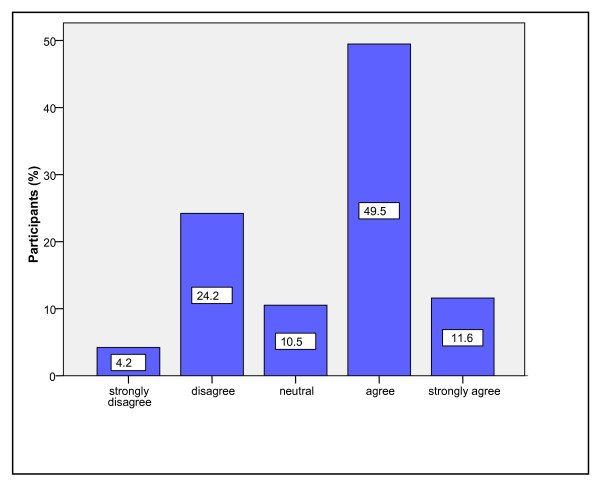
Percentage of participants by level of agreement to the statement,"In general the ED is adequately supervised"

**Figure 2:**
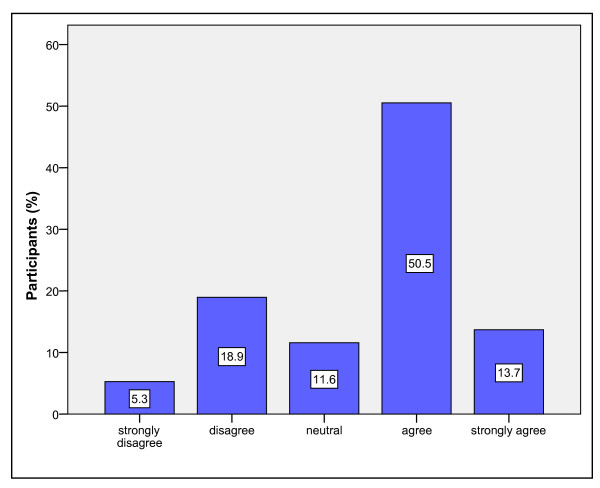
Percentage of participants by level of agreement to the statement, "In general the medical staff of the ED are adequately supervised"

Most participants (58/95, 61%) agreed/strongly agreed that the ED was, in general, adequately supervised (Figure 1). Similarly, the majority of participants agreed/strongly agreed (71/95, 64.2%) that the medical staff in their ED were adequately supervised (Figure 2).

In general, consultants and registrars were reported by participants as being more likely to provide supervision to interns in the ED; 66% (63/95 indicated that consultants provided most supervision to interns while 55% (52/95) indicated that most intern supervision was provided by registrars. These data were derived from separate questions.

**Figure 3:**
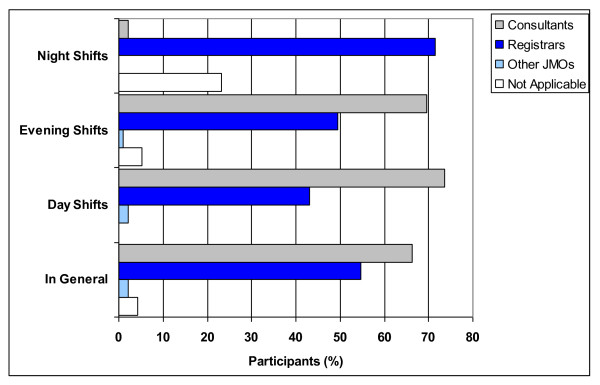
Percentage of participants according to perceived intern supervisory practices

The level of medical staff responsible for supervision of interns was found to vary depending on the shift (Figure 3). Participants reported that during the day and evening shifts, consultants were more likely to supervise interns than registrars. Registrars were, however nominated as being more likely to be responsible for the supervision of interns during night shifts, with 72% (68/95) of participants indicating that registrars were responsible and 2% (2/95) indicating that consultants were responsible during these shifts.

Most (64%) ED Directors and DEMTs agreed/strongly agreed that interns received adequate clinical supervision in the ED (Table 1). Participating emergency trainees and interns also mostly agreed/strongly agreed (79%) that this was the case. A similar result was obtained when analyses were restricted to interns only.

**Table 1:** Number and percentage of ED Directors and DEMTs by level of agreement regarding receipt of adequate clinical supervision by medical staff groups

Staff type	N	Strongly disagree	Disagree	Neutral	Agree	Strongly agree	N/A	Don't Know	Total
**Interns**	N	-	9	6	32	9	5	-	61
	%	-	14.8	9.8	52.5	14.8	8.2	-	100.0
**PGY 2, 3 or later**	N	1	14	3	38	4	1	-	61
	%	1.6	23.0	4.9	62.3	6.6	1.6	-	100.0
**Emergency trainees**	N	1	6	4	40	4	6	-	61
	%	1.6	9.8	6.6	65.6	6.6	9.8	-	100.0
**CMOs**	N	1	5	7	31	-	16	-	60
	%	1.7	8.3	11.7	51.7	-	26.7	-	100.0
**Pre-registration IMGs**	N	2	7	1	23	4	21	1	59
	%	3.4	11.9	1.7	39	6.8	35.6	1.7	100.0

Most ED Directors and DEMT also agreed/strongly agreed that adequate clinical supervision was provided to PGY 2,3 or later (69%), emergency trainees (72%) and CMOs (52%). Of those that had pre-registration IMGs on staff, most agreed that they were adequately supervised (Table 1).

When invited to comment about supervision of medical staff in their ED, 85 participants generated 90 comments [13 interns, 34 registrars, 34 ED directors, and 21 DEMT], subsequently categorised into seven themes (Table 2). While the modal response indicated that adequate supervision was in place this represented just 38.9% of responses; other responses were either negative with regard to the level of supervision or identified potential for improvement with more senior staff.

**Table 2:** Number and percentage of responses about supervision in the ED according to identified theme

Theme	Frequency of responses	Percentage of responses
**Adequate supervision processes in place**	35	38.9
**Level of supervision decreases during unsocial shifts**	14	15.6
**Other**	10	11.1
**Supervision is dependent on service demands**	10	11.1
**Supervision would improve with more senior medical staff**	9	10.0
**Overall, Supervision is inadequate**	7	7.8
**Supervision of pre-registration/middle level medical officers is overlooked**	5	5.6
**Total**	90	100.0

Some of the comments about supervision levels in the ED are listed below:

The most common theme with regard to supervision was that adequate supervision processes were in place, with several respondents indicating that this was facilitated by a formal or team-based structure. For example:

"It's a big ED, so having so many people means that you have to get scientific about supervision, have systems and rules in place (e.g. who is supervising who, one supervisor at a time). Not perfect but ok." (DEMT)

"We take supervision very seriously and have only recently revised supervision. We now have a specific person who has a very low patient load and as a result has a much more strengthened role in supervision and provision of advice and service. It is essential that the person who is in charge of the department and flow, has to provide a role where they have no significant patient load; it has taken a long time to get to this but we are starting now." (Director)

"[This hospital] is an exception in terms of the amount of supervision. Most other places I have worked at don't have this level of supervision, it can sometimes slow things down things but by and large we are vigilant, if things get busy it is the first thing that suffers, if teaching is included under supervision then it is the first thing to suffer, and they probably don't get the level that they should." (Registrar)

"We have a robust structure not dependent on individuals." (Director)

"I think our team based structure is really very good, because there is always an accountable officer, I have worked in EDs where it is every man for themselves and the doctors can work reasonably independently without any one realising that no-one is supervising them, so here it is a team based structure, the senior house officers are independent so it is the junior house officers and interns that need to be supported." (Registrar)

Several respondents felt that the level of supervision decreased during unsocial shifts

"The main issue here is that on night shifts the RMOs (e.g., PGY 2,3,4) are less supervised than they should be." (Director)

"During the day shifts [the ED] is well supervised, at night time there is a supervision process where a consultant is on call but sometimes the PGY 2 are in the ED by themselves while there is only one other support available - but at the same time that person might be busy dealing with something else." (DEMT)

"Not enough supervisors and too many people requiring supervision and increasing number of people who require really close supervision with our staffing numbers decreasing weekends are a real problem. Consultant cover is not as good as it used to be and we don't have consultant cover during the entire weekend." (Director)

Others felt that supervision was dependent on service demands

"Because of the level of overcrowding in the EDs and increased patient acuity, even though we try to provide large amounts of supervision, it is not consistent. We have a 30 minute rule: Junior staff have to discuss with senior staff within this time. Because of overcrowding, acuity etc this does not always happen." (Director)

"The ED is so busy and we are access blocked so the consultants have to sort out the main part of the ED and supervise the flow so there really isn't enough time to adequately teach and supervise the junior medical staff, to give them the appropriate attention that each doctor deserves. It is very hard for a consultant to run an ED and try and teach someone when it is absolute chaos." (DEMT)

"It is flow dependent, when it is quiet the supervision is better. When busy and senior staff are distracted by multiple nursing requests for assistance and by resuscitation cases or very unwell patients ... the supervision falls off because there is no designated person for supervision and people are left by themselves really, and this is were it falls down.. What happens during these times is that you have more access block because no decisions are being made or patients are given, what we would consider poor advice and the patients just vegetate for hours waiting for something to happen so you have to be very on the ball about grabbing junior doctors at times when it is busy and saying ‘tell me in 5 word or less what is happening and I will tell you what to do'." (Registrar)

Supervision comments of interns versus more senior medical staff

When examined according to participant type, responses provided by interns most commonly suggested that an adequate supervision process was in place (9/13 responses), whereas those provided by registrars were generally split between those who felt an adequate supervision arrangement was in place (6/22) and those who felt supervision fluctuated according to service demands (6/22). While response by ED Directors and DEMT were spread more across themes, the modal theme for each of these participants types was that an adequate supervision process was present (DEMT, 7/21; ED Directors 13/34).

### Feedback

All ED Directors and DEMTs from sites that had interns indicated that interns received formal feedback during their ED rotation (Table 3). The majority (30/33, 91%) of emergency trainees and interns also agreed/strongly agreed that they received feedback on their ED Rotation. This was also the case when analyses were restricted to interns only with 14/15 interns agreeing.

**Table 3:** Number and percentage of ED Directors and DEMT by level of agreement to statements indicating that feedback is provided to medical staff

Staff Type	N	Strongly disagree	Disagree	Neutral	Agree	Strongly agree	N/A	Don't Know	Total
**Interns**	N	0	0	0	30	26	5	0	61
	%	0.0	0.0	0.0	49.2	42.6	8.2	0.0	100
**PGY 2, 3+**	N	1	1	5	37	16	1	0	61
	%	1.6	1.6	8.2	60.7	26.2	1.6	0.0	100.0
**Advanced trainees**	N	0	0	0	25	30	6	0	61
	%	0.0	0.0	0.0	41.0	49.2	9.8	0.0	100.0
**CMOs**	N	4	19	3	15	3	14	0	58
	%	6.9	32.8	5.2	25.9	5.2	24.1	0.0	100.0
**Pre-reg. IMGs**	N	0	3	0	22	12	18	1	56
	%	0.0	5.4	0.0	39.3	21.4	32.1	1.8	100.0

Most ED Directors and DEMTs agreed that PGY 2,3 or later, emergency trainees and where applicable, pre-registration international medical graduates (IMGs) received formal feedback in the ED. Responses varied with regard to feedback provision to CMOs, slightly more disagreed/strongly disagreed that formal feedback was provided (23/61 37.7%) than those who agreed/strongly agreed (17/61, 27.9%) that feedback occurred (Table 3).

Seventy-five respondents provided a total of 95 responses regarding the ability to provide feedback to junior staff in the ED [13 interns, 22 registrars, 33 ED directors, and 27 DEMT]. These were categorised post-hoc into one of nine themes (Table 4). Inter-rater reliability of thematic coding was adequate (κ =.70).

**Table 4:** Number and percentage of responses regarding the ability to provide feedback to junior staff in the ED according to thematic content

Theme		Number of responses	Percentage of responses
	**Formalised/structured approach to feedback provision**	34	35.8
	**Informal Feedback provided**	1	1.1
	**Service requirements restrict ability to provide feedback**	10	10.5
	**Feedback concentrates on those who are not meeting standards**	4	4.2
	**Feedback process is inadequate**	14	14.7
	**Pre-registration/middle level medical staff are overlooked**	1	1.1
	**Good Feedback**	23	24.2
	**Consultant driven feedback**	4	4.2
	**Other**	4	4.2
	**Total**	95	100.0

The most common response regarding feedback to junior medical staff was that a formalised or structured approach was adopted for feedback provision

"we have a meeting once a month where all consultants can provide feedback and a designate supervisors provides feedback in an interview as well - it is multi-sourced feedback which I think is quite good." (DEMT)

*"We have a performance management tool that is being developed by the ED director and the hospital so everybody is performance managed and gets feedback." (DEMT*)

"We divide all trainees and non-trainees amongst the consultants so we have dedicated time in our admin time for feedback we discuss them at a senior meeting and it is then feedback to staff, mid term and end of term assessment, as the DEMT I do all of the people on the training scheme." (DEMT)

"What I do is a 360 degree assessment, I go to consultants, registrars, to get an appraisal and then I combine it together and sit down with the junior staff member to provide them with the feedback." (Director)

Several respondents also indicated that the quality of feedback provided was good

"Generally it is really good, you get a lot of informal feedback as you go along but I guess as part of an internship you have to get a performance review every rotation, it depends on your consultant, mine has been really supportive and sort of sat down with me and said you have been really good, could improve here and there, but it was really good." (Intern)

Others felt the feedback process was inadequate or inappropriate

"There are not enough tools to use for feedback and so on, I would like more training about how to give feedback for staff, you can get frustrated in ED and just tell them off, people need to be trained and skilled to do this in the right way" (Director)

"Insufficient - all tied up with the insufficient staff numbers" (DEMT)

I think the current form is flawed, the form is a duffer, there are 2 problems; one is that you don't work with the same person every shift, the supervisors change all the time so what we have implemented over the last couple of years is for each JMO to have a mentor and they provide a commentary on how they perform at a departmental, interpersonal, documentational level and whatever else is required on the form. This is a strategy to try and improve the sensitivity of the observations rather than having the director ticking forms at the end of semester." (DEMT)

"I think that a lot of the feedback when we raise concerns is like a toothless tiger in that you represent your concerns clinically and not a great deal is done about it, in that the system is not built to rehabilitate the flaws of medical staff so the feedback can be a largely pointless... if we raise a clinical concern or something which has not gone well then there is not a lot of support for these circumstances." (DEMT)

Some felt that service requirements restrict the ability to provide feedback

"It's made significantly more difficult due to significant overcrowding and access block, so it does impact on all of these things, supervision of medical staff in the ED, it has sort of far reaching impacts about workplace flow and work practices which I think impacts on all of those areas which I think would be the primary problem and while we have good set ups in regard to education and supervision, they are all made much more difficult because of overcrowding and access block" (DEMT)

### Feedback comments of interns versus more senior medical staff

When analysed according to participant groupings, the most common responses by interns, registrars and directors regarding feedback were split between two main themes: a structured or formal approach being in place (interns, 4/13; registrars,6/22; Directors 12/33) and fluctuation in the provision of feedback due to service demands (interns, 4/13; registrars 5/22; Directors 10/33). The dominant themes expressed by DEMT, however was that a structured or formal approach to feedback was present (12/27) with other responses spread broadly across other themes.

## Discussion

The EMCAS study identified a number of issues related to junior medical staff supervision and feedback. The majority of staff interviewed indicated that, in general, Australian interns were adequately supervised. Despite the potential for bias from a sample with more supervisors, interns more commonly offered the view that adequate supervision arrangements were in place than more senior medical staff, whose responses more often suggested these processes were influenced by service demands. Contrary to findings from previous studies, consultants had the major supervisory role apart from overnight, when registrars took most of this responsibility. This may be a function of the continued development of the specialty of emergency medicine in Australasia, and the rapid growth in consultant numbers, currently standing at 1250. It may now be possible for consultants, previously constrained by service commitments, to undertake effective supervisory roles, just on the basis of improvements in senior staffing.

Many staff felt that there were good processes in place to ensure adequate supervision, facilitated by a formal or team-based structure. There was a common view that a systematic approach to supervision that was not dependent on individuals worked best, although that required clear departmental and hospital support in freeing up senior staff from their patient loads. Again, this may have become possible through the growth in the workforce in emergency medicine in Australia.

Both feedback and supervision of interns were reported to fluctuate with the service demands of EDs, and with time of day and day of week. Weekends and night shifts were felt to be particularly prone to inadequate supervision. Service demands represent the greatest threat to the provision of adequate supervision of interns working clinically. Innovative projects like the More Learning for Interns in Emergency (MoLIE) initiative by Queensland Health may address this issue. In MoLIE, interns are taught "off the floor" for 20% of their training time (or roughly two half days per week) by dedicated funded consultant emergency physicians, reducing the burden of clinical supervision requirements in busy EDs and improving the educational experience for interns.

Qualitative data indicate that the greatest impediment to both adequate supervision of junior medical staff and the provision of appropriate feedback is overcrowding related to access block, the term used to denote a lack of inpatient bed access for patients admitted and waiting for a bed in the ED. Access block has a broadly detrimental effect on ED performance and is known to worsen ED mortality [10,11]. It is of concern that it is also likely to negatively affect supervision and junior staff feedback, and part of this mortality effect may be mediated by reduction in effective supervision due to overcrowding. While the effects of overcrowding and access block on quality of care are well documented [10-12], further research is required to elucidate these effects on teaching, learning, and supervision of junior medical staff.

While several staff reported having structured feedback in place, qualitative data from participants at all levels indicated room for improvement in this area, with a number of staff feeling that feedback mechanisms and processes were inadequate or inappropriate. Of all staff types, Career Medical Officers (CMOs) were perceived as having the poorest opportunities for supervision and feedback. CMOs represent a small group of staff that have a variable presence in EDs and who may have a significant amount of experience in the ED context. In many EDs, they are highly valued, particularly where there are shortages of junior staff. It is apparent, however, that CMOs are somewhat overlooked with regard to supervision and feedback, possibly as a result of their perceived seniority. Further research exploring the educational experience of CMOs in the ED is warranted.

## Limitations

This study was limited by the low participation by interns and emergency trainees, which may have biased views towards those of supervisors. Analysis of comments about supervision and feedback however failed to reveal such bias, with interns more likely to say structured supervision was in place than more senior doctors.

Due to privacy constraints and difficulties associated with obtain contact information for this disparate group of participants, we relied on the secondary distribution of survey invitations by site co-ordinators. Although a representative sample can be obtained despite low participation, we cannot exclude the possibility that our sample were not representative of the full intern and emergency trainee population. It is however, important to acknowledge that the sample of interns and registrars was drawn from numerous hospital sites.

As this study formed part of a larger project assessing the capacity for EDs to absorb an increased number of interns and medical students, the imbalanced participation may also be attributable to a high level of motivation by ED Directors and DEMTs to voice their concern due to both a greater level of awareness of the anticipated increase combined with their management roles.

While most participating registrars and Directors/DEMTs would have had supervisory roles by virtue of their senior positions, we did not determine the extent to which these staff undertook formal supervision of interns or other staff nor have we contrasted the viewpoints of junior medical staff participants with their direct supervisors. This may have resulted in an imbalanced viewpoint. The perceptions of supervisors have received little attention in the medical literature and are worthy of future consideration.

The term "adequacy" was not defined by the researchers; participants responded according to their own views of what adequate meant. This may have introduced some ambiguity. Finally, the survey instrument had not previously been validated.

## Conclusion

Consultants appear to provide the majority of supervision of junior medical staff in Australian EDs, and supervision is generally felt to be adequate by all groups, although constrained at times by service provision. While feedback is also felt to be adequate, there appear to be deficiencies in feedback mechanisms, and CMOs particularly are overlooked. Further research quantifying the exact role and extent of senior medical staff in the supervision of junior medical staff is suggested. Further, the effect of access block and overcrowding on supervision requires more detailed study.

## Competing interests

The authors declare that they have no competing interests.

## Authors' contributions

GAJ was involved in the conception and design of the study, and analysis and interpretation of data; drafting the manuscript and revising it critically; and gave final approval of the version to be published. TJW was involved in the conception and design, and analysis and interpretation of data; drafting the manuscript and revising it critically for important intellectual content; and gave final approval of the version to be published.  CM was involved in acquisition, analysis and interpretation of data; drafting the manuscript; and gave final approval of the version to be published.

## Pre-publication history

The pre-publication history for this paper can be accessed here:

http://www.biomedcentral.com/1472-6920/10/74/prepub

## References

[B1] Graham IS Gleason AJ Keogh GW Australian Curriculum Framework for Junior Doctors Med J Aust 2007 186 S14 19 17407415 10.5694/j.1326-5377.2007.tb00959.x

[B2] Sox CM Burstin HR Orav EJ The effect of supervision of residents on quality of care in five university-affiliated emergency departments Acad Med 1998 73 776 782 10.1097/00001888-199807000-00017 9679467

[B3] Hore CT Lancashire W Fassett RG Clinical supervision by consultants in teaching hospitals Med J Aust 2009 191 220 222 19705984 10.5694/j.1326-5377.2009.tb02758.x

[B4] Dent AW Crotty B Cuddihy HL Learning opportunities for Australian prevocational hospital doctors: exposure, perceived quality and desired methods of learning Med J Aust 2006 184 436 440 16646742 10.5694/j.1326-5377.2006.tb00314.x

[B5] Dale J Williams S Wellesley A Training and supervision needs and experience: a longitudinal, cross-sectional survey of accident and emergency department senior house officers Postgrad Med J 1999 75 86 89 10448468 10.1136/pgmj.75.880.86 PMC1741120

[B6] Lack CS Cartmill JA Working with registrars: a qualitative study of interns' perceptions and experiences Med J Aust 2005 182 70 72 15651964 10.5694/j.1326-5377.2005.tb06578.x

[B7] Paltridge D Prevocational medical training in Australia: where does it need to go? Med J Aust 2006 184 349 352 16584371 10.5694/j.1326-5377.2006.tb00270.x

[B8] St Vincent's Health Emergency medicine capacity assessment study. Final report to the Commonwealth of Australia Department of Health and Ageing. Melbourne 2009

[B9] Ritchie J Spencer L Hubermann AM, Miles MM Qualitative data analysis for applied policy research The Qualitative Researcher's Companion An Expanded Sourcebook 2002 2 Thousand Oaks, California: Sage Publications 305 330

[B10] Richardson DB Increase in patient mortality at 10 days associated with emergency department overcrowding Med J Aust 2006 184 213 216 16515430 10.5694/j.1326-5377.2006.tb00204.x

[B11] Sprivulis PC Da Silva JA Jacobs IG The association between hospital overcrowding and mortality among patients admitted via Western Australian emergency departments Med J Aust 2006 184 208 212 16515429 10.5694/j.1326-5377.2006.tb00416.x

[B12] Richardson DB Mountain D Myths versus facts in emergency department overcrowding and hospital access block Med J Aust 2009 190 369 374 19351311 10.5694/j.1326-5377.2009.tb02451.x

